# Evaluation of Fenugreek (*Trigonella foenum-graceum* L.), Effects Seeds Extract on Insulin Resistance in Women with Polycystic Ovarian Syndrome

**Published:** 2013

**Authors:** Maryam Hassanzadeh Bashtian, Seyed Ahmad Emami, Nezhat Mousavifar, Habib Allah Esmaily, Mahmoud Mahmoudi, Amir Hooshang Mohammad Poor

**Affiliations:** a *Department of Midwifery, School of Nursing and Midwifery, North Khorasan University of Medical Sciences, Bojnurd, Iran. *; b *Department of Pharmacognosy, School of Pharmacy, Mashhad University of Medical Sciences. Mashhad, Iran. *; c *Department of Obstetrics and Gynecology, School of Medicine, Mashhad University of Medical Sciences. Mashhad, Iran. *; d *Department of Biostatistics, Faculty of Health, Mashhad University of Medical Sciences. Mashhad, Iran. *; e *Department of Immunology, School of Medicine, Mashhad University of Medical Sciences. Mashhad, Iran. *; f *Department of Pharmacology, Mashhad University of Medical Sciences. Mashhad, Iran. *

**Keywords:** Fenugreek, Insulin resistance, Metformin, PCOS, Oligo-Amenorrhea

## Abstract

PCOS (Polycystic Ovarian Syndrome) is associated with insulin resistance, obesity and disorders of lipid metabolism as well as infertility. Fenugreek seeds extract is successfully used in lowering blood glucose. Metformin has also the same effect but in a different way. The aim of this study was the assessment of fenugreek effects on insulin resistance in women with PCOS**. **

This was a prospective randomized, double-blind, placebo-controlled trial. The study was conducted at the Montaserieh Hospital in Mashhad University of Medical Sciences, Mashhad, Khorasan Razavi Province, Iran. The patient population included 58 oligo-anovulatory PCOS women with typical ovaries. Women were randomly allocated to receive hydroalcoholic extract of fenugreek seeds in capsules with metformin (n = 30) or placebo capsules with metformin (n = 28) and were assessed before and every 4 weeks within a treatment period of 8 weeks.

Menstrual disturbance and metabolic parameters (markers of insulin resistance and hormonal parameters) were measured. Insulin resistance based on HOMA-IR (homeostasis model assessment for insulin resistance) model was not significantly different between two groups. Ultrasound scans were performed before and at the end of 8 weeks treatment with significant decrease in PAO (polycystic appearing ovaries) in group 1 (p = 0/01).

Adjuvant therapy to the fenugreek seeds extract (with metformin) in PCOS women improved the sonographic results and menstrual cyclicity.

## Introduction

PCOS (polycystic ovary syndrome) is the most common hormonal disorder among the women of reproductive age, affecting 5-10% of the population (1). According to AES (Androgen Excess Society) diagnostic criteria, PCOS is distinguished by at least two of the following key factors: The existence of hyperandrogenism clinical (hirsutism) and/or biochemical and either oligo or anovulation and finally presence of polycystic ovarian morphology (2). 

In many women with PCOS, insulin resistance, obesity, disorders of lipid metabolism and infertility can also be found. Hyperinsulinemia may increase abnormal ovarian androgen secretion and develop abnormal follicles, leading to dysfunctional ovarian and menstrual cyclicity (1).

Metformin has been showed as the first choice treatment in the therapy for reproductive disorders caused by PCOS (3). Metformin inhibits hepatic gluconeogenesis, and also, has proved to have a direct effect on ovarian steroidogenesis in granulosa cell cultures (3). Metformin treatment can cause improvements in insulin resistance and ovarian hyperandrogenism in women with PCOS (4). 

In recent years, the role of complementary medicine approaches has become very trendy (5). Fenugreek (Trigonella foenum-greacum L.) is an herbal drug (6). The seeds of fenugreek have been reported to have anti-diabetic and hypocholesterolemic effects in both animal models and humans (7). 

Fenugreek administration has not been reported to cause any toxicological effects (7). In addition, according to Shamas *et al. *study, it is safe to use Fenugreek (8). This trial was planned to assess the effects of Fenugreek (Trigonella foenum-greacum L.) seeds extract on insulin resistance in women with PCOS. 

## Experimental

*Study population*

Women with PCOS (n = 58) aged 20-35 years, whose chief complaints were menstrual disturbances and infertility and/or clinical signs of hyperandrogenism (*e.g. *hirsutism and acne) were enlisted between 2007 and 2009 from the Montaserieh Hospital (Mashhad, Iran). The study protocol was approved by the Ethical Committee of the Mashhad University of Medical Sciences, and written informed consent was obtained from all patients. The diagnosis of PCOS was based on at least on this criteria that all patients were expected to have disrupted ovulatory function with chronic oligomenorrhea (cycle length > 35 days) or amenorrhea (cycle length > 12 weeks) and typical appearance of polycystic ovaries by ultrasound according to the criteria of the Rotterdam consensus meeting 2003 (9, 10) and AES (3), which hyperandrogenism (serum free testosterone (T) concentration 7.01 ≥ pg/mL). The BMI (body mass index) was not considered as inclusion or exclusion criterion. The presence of the following disorders was excluded by specific laboratory tests: 

Any form of diabetes mellitus, hyperprolactinemia, thyroid disorders, late onset congenital adrenal hyperplasia (exclusion of 21 hydroxylase deficiency by molecular genetic analysis). 

None of the women had taken any anti-obesity medications during 6 months before inclusion in the study. We confirmed the absence of heart, liver, or kidney diseases (predisposing lactic acidosis) and unsuspected pregnancy in all participants before the inclusion in this study. 

*Preparation of fenugreek capsules*

Dried and fresh fenugreek seeds (25 Kg) were obtained from a commercial source. Seeds were washed in distilled water and were ground to a fine powder in a mixer under chilled conditions. Obtained powder was extracted by percolated white ethanol (70% w/w). The ethanol extract was lyophilized. The lyophilized extract was 2600 g. Twenty-five percent of tricalcium phosphate was added to dried extract. The powder mixture was crushed in a blinder and passed through a 20- mesh sieve. A capsule-filling machine was used for preparation of powder containing capsules. The mean weight of each capsule was 525 mg (500 mg dived extract). Placebo capsules were filled (525 mg) with lactose powder colored with 1% tartrazin. 

*Study design*

Patients were randomly allocated to one of the two groups. The first person entering the study was assigned to either drug group (group 1) or placebo (group 2) by chance. The rest were assigned one by one to either of the groups. Group 1 (n = 30) received metformin plus fenugreek seeds extract, group 2 (n = 28) received placebo plus metformin. Fenugreek seeds extract and placebo were provided by Goldarou Pharmaceutical Co. Isfahan, Iran. Metformin was administered at a dosage of 3 500 × mg daily, except for the first week of treatment when 500 mg was at first given once a day for three days and then twice a day for other three days to reduce the incidence and severity of gastrointestinal side effects.

Randomization was done in a prospective, placebo-controlled; double blind patients received fenugreek seeds extract plus metformin or placebo plus metformin. Fenugreek seeds extract was administered at a dosage of 2 × 500 mg daily and also placebo in group 2 for breakfast and dinner. 

The coded drugs were given to the patients by pharmacist at Montaserieh Hospital. The randomization code was not broken until the last patient completed all observations. 

Patients were advised to use barrier contraception if fertility was not desired and were carefully instructed to stop taking the drug immediately on confirmation of pregnancy. 

All patients underwent clinical, metabolic, and hormonal evaluations at baseline and in regular intervals of 4 weeks during the whole treatment period of 8 weeks after randomization. In the baseline study, vaginal bleeding was induced by progesterone withdrawal or spontaneous bleeding. Clinical assessment included menstrual cycle frequency, height, weight, BMI and hirsutism. The following studies were performed on the 2^nd^ till the 5^th ^day of their period cycle: After a 12-h overnight fasting, a nonheparinized venous blood sample was obtained from 08:00 to 09:00 to measure the circulating concentrations of prolactin, LH(luteinizing hormone), FSH (follicle stimulating hormone), free testosterone, TSH (thyroid stimulating hormone), DHEA-S (dehydroepiandrosterone sulfate), 17 *α *-OHP (17 *α *-hydroxyprogesterone), fasting glucose and insulin, SGPT, SGOT, and renal chemistry were ascertained to recognize and prevent possible metformin-induced complications. 

After obtaining the basal blood sample, a 2-h OGTT (oral glucose tolerance test) was immediately performed with an oral glucose load of 75 g, and nonheparinized blood samples were obtained after 120 min to measure serum glucose concentration. 

Every 4 weeks during the 2-month study period, the baseline clinical metabolic (including the OGTT, fasting glucose and insulin) and hormonal evaluations were performed (except LH, FSH, free testosterone and 17α -OHP, which were reassessed only at the end of the study period) or on 2-5 days of a spontaneous menstrual bleeding. 

Improvement of cycle disorders was defined as a change among clinically classified cycle groups (amenorrhea/oligomenorrhea/ eumenorrhea) or a reduction in cycle length to at least 4 weeks or occurrence of pregnancy. 

BMI was calculated using the equation of weight (Kg/m^2^). Hirsutism was clinically evaluated using the F-G (Ferryman-Gallwey) score (assessment of 11 body areas); a score greater than 8 was defined as hirsutism (11). 

Using the serum glucose and insulin concentrations during fasting and the 2-h OGTT, we calculated the following parameters: 

A. FGIR (fasting glucose to insulin ratio) (1) = fasting serum glucose concentration (mg/dL) / fasting serum insulin concentration (microinternational units per mL). 

B. HOMA-IR (homeostasis model assessment for insulin resistance) (2, 12-14) = fasting serum insulin (microinternational units per mL) × fasting serum glucose (mmole/L) / 22.5. 

C. QUICKI (Quantitative insulin sensitivity check index) (12-14) =1/ [log (I0) + log (G0)], where I0 = fasting serum insulin (microinternational units per milliliter) concentration and G0=fasting serum glucose (milligrams per deciliter) concentration. 

D. *β *cell function index (15, 16) = (20 × fasting serum insulin (microinternational units per mL) concentration / fasting serum glucose concentration (mmole/L) - 3.5. 

Concentrations of FSH, LH, TSH, PRL, free testosterone, DHEAS, insulin and 17 *α *-OHP were analyzed using BioSource immunoradioactive assay kits (BioSource Europe SA, Belgium) and analyzed on GAMMATic gamma counting system (kontron, Switzerland). These previously mentioned assays were performed in the endocrine laboratory of the Special Medical Laboratories (Imam Reza Hospital, Mashhad University of Medical Sciences, and Mashhad, Iran). 

Plasma glucose levels, Cr, SGOT, SGPT were assayed by the Pars Azmoon kits (Tehran, Iran) on automated chemistry analyzer (Alcyon 300i (Abbott Inc, Abbott pork, IL)).

The pharmaceutical company providing the drugs was not involved in the study design, expenses, data collection, data analysis, data interpretations or writing the report. No funding of any kind was received to perform the study or by any of the participants in the study.

*Statistical analysis*

All data presented as median and interquartile (1-3 quartile) unless otherwise noted. Descriptive statistics were used for continuous data at each visit. Treatment groups, insulin resistance, their interaction, and the age of the patients were chosen as covariates. Changes in parameters over time were assessed using repeated measurement ANOVA, and differences between groups were evaluated using a nonparametric test (Mann-Whitney). Within-group differences were analyzed by the paired Student›s t-test or Wilcoxon test/sign test. P < 0.05 was considered to indicate statistical significance. *SPSS software (Statistical Product and Services Solutions, version 13.0, SPSS Inc, Chicago, IL, USA) was used to analyze the data*.

## Results and Discussion

A total of 58 patients were recruited to the study but 13 patients in first month, 4 patients in second month and 7 patients in the end of treatment were withdrawn from the study for reasons unrelated to the use of fenugreek. Three of the patients became pregnant during the first (two fenugreek/one placebo) month of treatment.

 Treatment compliance was good and most of the patients did not report any drug related adverse effects.

BMI was between 21.19 and 45.73 Kg/m^2^, median 30.36 Kg/m^2^. Some patients dropped out of the study in case of diagnosis of pregnancy or because of loss of follow up.

No significant difference in clinical baseline characteristics could be observed between the two groups (Table 1). During 2 months of treatment with fenugreek, no significant change could be observed in body weight or BMI. Ultrasound scans were performed before and after 2 months of treatment with significant decrease in PAO (polycystic-appearing ovaries) (p = 0.01). The F-G score remained unchanged in both groups after 2 months of treatment.

**Table 1:** Clinical and sonographic characteristics of the patients

**Parameter**	**Drug (n = 23)**		**Placebo(n = 22)**		
	Baseline	8wk	Baseline	8wk	P
Age,yr	25(23-31)		25(23.75-26.25)		ns
Age of menarch,yr	14(12-14)		14(13-15)		ns
Weight,Kg	70(65-75)	68(63-78)	78(68-84.62)	73.5(65.25-81.87)	ns
BMI,Kg/m^2^	28.67(27-31.23)	29.04(26.77-31.4)	31.99(28.37-35.34)	30.51(27.8-33.59)	ns
Hirsutism,F-G Score	9(6-14)	8(6-12)	9(7-12)	9(7-12)	ns
Duration of married,yr	6.5(3.5-9)		6(3.5-7)		ns
Duration of infertility,yr	4.5(2-7)		5(3.25-6.25)		ns
PAO(%)	100	0	100	53/8	s

Ns: Not significant, S: Significant

Before the treatment, no women had regular menses. 24/22 (fenugreek/placebo) women were oligomenorrheic and 7/5 were amenorrheic. After 2 months of treatment, 12/21women got their cycles totally restored to eumenorrhea (cycle length = 28 ± 5 days), 6/11 women were oligomenorrheic, and 2/6 women still had amenorrhea. Most of the subjects were converted only one step (*e.g. *amenorrhea to oligomenorrhea); a two step conversion was rare.

Twenty-eight women were found to have insulin resistance: 15 patients of the placebo group/13 patients of the fenugreek group.

None of the women showed any degree of glucose intolerance at baseline as this was an exclusion criterion. At baseline, metabolic parameters were comparable in both groups. All measures of insulin-related parameters, like FGIR, HOMA-IR, QUICKI and *β*-cell function, were similar in both groups. There was also no difference in levels of fasting glucose and fasting insulin. Markers of insulin resistance (HOMA-IR) and insulin sensitivity (QUICKI) did not show any change with statistical significance in both groups. Tables 2 and 3 are present metabolic parameters.

**Table 2:** Metabolic parameters (Drug

	**Baseline**	**4 weeks**	**8 weeks**
Fasting glucose (mg/dL)	85(78.75-90.25)	83(78-92)	82(78-93.5)
Fasting insulin (μu/mL)	9.9(8.2-14.25)	15.75(9.67-18.37)	14(10.6-19.75)
OGTT	106(96-114)	98(84-129)	107(92.5-139.5)
HOMA-IR	2.06(1.75-2.85)	2.93(1.74-4.1)	3.09(2.16-4.05)
FastingG/I ratio	7.87(5.89-10.95)	5.65(4.86-8.38)	5.92(3.95-8.32)
QUICKI	0.34(0.32-0.35)	0.32(0.31-0.35)	0.32(0.31-0.34)
*β*-cell function	42.23(29.40-57.91)	60.21(40.01-70.57)	57.27(40.6-87.73)

Values are median and 1-3 quartiles

**Table 3:** Metabolic parameters (Placebo

	**Baseline**	**4 weeks**	**8 weeks**
Fasting glucose (mg/dL)	91(85-98)	85(78.25-97.25)	91(85.75-95.75)
Fasting insulin (μu/mL)	9(6.8-23)	13.25(6.5-19.07)	16.7(11-23)
OGTT	103(90.2-116.5)	114(89-134.7)	107(83-156.7)
HOMA-IR	1.98(1.54-5.94)	2.64(1.27-4.17)	3.58(2.26-5.06)
FastingG/I ratio	9.55(4.1-13.43)	7.1(5.15-12.34)	6.08(5.03-8.49)
QUICKI	0.34(0.39-0.35)	0.33(0.3-0.37)	0.31(0.3-0.33)
*β-*cell function	34.2(23.31-84.26)	47.89(25.7-66.46)	56.01(38.93-69.01)

Values are median and 1-3 quartiles

Table 4 presents hormonal parameters. The baseline hormone concentrations were similar in both groups. Thirty-three of the 58 subjects (56.8%) had hyperandrogenism (increased F-G score and/or increased serum T). Circulating androgen concentrations and 17-*α *OHP levels remained unchanged throughout the whole study period. In contrast, basal LH and FSH were slightly increased at the end of the trial only in the placebo group.

**Table 4:** Hormonal parameters (Drug and placebo

**Parameter**	**Drug**		**Placebo**	
	**Baseline**	**8 weeks**	**Baseline**	**8 weeks**
FSH(IU/L)	4.5 (3.7-5.2)	5.4 (4.15-6.5)	4 (3.1-5)	6.3 (5.1-7)
LH(IU/L)	8.7 (6-12.6)	10.2 (6.37-11.52)	5.85 (5-8)	8.2 (6.6-15.1)
17OH PG(ng/mL)	1.5 (1.1-1.95)	1.6 (0.9-3.75)	1.2 (0.95-1.62)	2 (1.4-3.5)
Free testosterone(pg/mL)	3.9 (1.95-17.4)	3.6 (0.9-6.05)	2.2 (1.3-5.9)	2.9 (1.2-5.4)

Values are median and 1-3 quartiles

To our knowledge, this is the first prospective, randomized , double-blind, placebo-controlled trial to address the issue of additive effect of fenugreek seeds extract plus metformin in the treatment of cycle disorders as primary outcome measure depending on the insulin resistance in women with PCOS.

In recent years, the role of alternative therapy approaches has become very popular (5). In this study, treatment with fenugreek seeds extract plus metformin in comparison with placebo plus metformin resulted in significant improvements in PAO and cycle disorders that these results are in agreement with the reported finding by Eiesenhardt *et al. *They have shown to improve the menstrual cyclicity and ovulatory function in the group that was just treated with metformin (2).

Fleming study also showed that metformin treatment improves the ovulation function in women with oligo menorrhea and polycystic ovaries. In Fleming *et al. *no changes in fasting glucose concentrations, fasting insulin or insulin responses to glucose challenge was recorded after 14 weeks of metformin or placebo therapy (17) that was similar to our results.

Al-Habori showed that fenugreek administration may increase plasma insulin levels *in-vivo *(7). Despite of the similarity between his results and ours, our study did not produce any significant results.

Sharma, observed a significant improvement in plasma glucose and insulin responses in his subjects (18), although it was in contrast to our results.

Gupta *et al. *study results showed that in their treated group (received 1 g/day hydro alcoholic extract of fenugreek seeds) compared to control group (received placebo capsule and usual care) at the end of two months of treatment, fasting blood glucose and 2 h post glucose blood sugar were not significantly different, similar to our study results. But in their study, insulin was significantly lower (p < 0.001). HOMA model derived insulin resistance showed a decrease in percentage of beta-cell secretion in treated group as compared to control group and increase in percentage of insulin sensitivity (p < 0.05) (19), while in our study these data were not statistically different.

According to the Puri *et al. *treatment by water extract of seed of fenugreek produced significant attenuation of the glucose tolerance curve and improvement in the glucose-induced insulin response that the hypoglycemic effect may be mediated through stimulating insulin synthesis and/or secretion from the beta pancreatic cells of Langerhans (20). Results of Basch *et al. *study was similar to Puri study and there was also fenugreek seeds extract hypoglycemic effects by inhibiting the activities of alpha-amylase and sucrose, two intestinal enzymes involved in carbohydrate metabolism (21), and also it reduces the insulin resistance (22).

In Pirwany *et al. *study, the subgroup with raised fasting insulin showed less marked changes (23). In contract with the result of Harborne *et al. *(24) and Eisenhardt *et al. *(2) studies, it was showed that metformin did reduce markers of insulin resistance.

There has been no study about the fenugreek effect on hormonal levels (LH, FSH, free T, 17OHP). We observed no difference between the results obtained before and after the treatment. Our results are in agreement with the reported findings by Esenhardt except for that in LH level of their study, slight increase in the first month of the study was showed in both groups and was still seen high at the end of the trial only in the placebo group (2). But in Diamanti-kandarakis study, treatment with metformin was led to significant changes in sexual hormone levels (4).

In most cases, the trend for insulin levels to fall in women with PCOS or in other patient with diabetes in response to fenugreek and/or metformin has been confirmed by others. The non-significant decrease in insulin levels in this study was probably due to the fact that all patients were not markedly hyperinsulinaemic at the beginning and also may be due to the drop out in sample size.

However, our findings support the hypothesis that fenugreek seeds extract plus metformin, as compared with metformin plus placebo, didn’t have significant effect on insulin resistance.
